# Two-Dimensional Carrier Distribution in Top-Gate Polymer Field-Effect Transistors: Correlation between Width of Density of Localized States and Urbach Energy

**DOI:** 10.1002/adma.201303060

**Published:** 2013-10-30

**Authors:** Auke J Kronemeijer, Vincenzo Pecunia, Deepak Venkateshvaran, Mark Nikolka, Aditya Sadhanala, John Moriarty, Monika Szumilo, Henning Sirringhaus

**Affiliations:** 1 Cavendish Laboratory, University of CambridgeJ J Thomson Avenue, Cambridge, CB3 0HE, United Kingdom E-mail: ajk76@cam.ac.uk, hs220@cam.ac.uk

**Keywords:** organic electronics, field-effect transistors, carrier distribution, disorder, density of states, urbach energy

Organic electronics is becoming a viable technology in order to realize large-area and low-cost electronics.^1^,^2^ In order to make progress in the performance of organic circuitry, an improved understanding of the fundamental device physics of organic field-effect transistors (FETs) is a prerequisite. Accurate models for charge transport are needed for the development of device models that can be used as input for the design of integrated circuits. Organic FETs operate by accumulation of charge at the interface between the semiconductor and dielectric upon the application of a gate voltage.^3^ Charge transport from source to drain electrodes occurs solely in this thin sheet buried within the transistor structure. Although central to transistor operation, the extent of the interfacial accumulation layer into the bulk of the semiconductor and the detailed carrier distribution in the direction normal to the interface is poorly understood.

Experimentally, the extent of the accumulation layer has been inferred from measurements probing the performance of FETs as a function of semiconductor layer thickness. The mobility of charge carriers in thin sexithiophene (6T) crystals was shown to be optimal for crystal thicknesses of 3 monolayers,^4^ while the mobility saturated after two monolayers in polycrystalline 6T films.^5^ The mobility of evaporated pentacene films was shown to saturate after 6 monolayers.^6^ In situ measurements of source-drain current versus layer thickness in evaporated 6T have demonstrated a saturation of mobility in the range from 2–7 monolayers depending on the specific growth conditions.^7^ Similar measurements derived accumulation layer thicknesses of 9 nm for 6T and 2 nm for pentacene,^8^ while measurements of dihexylquaterthiophene (D4HT) yielded maximum mobility values for completed first and second monolayers while saturating for higher semiconductor coverage.^9^ Recently, using solution-based processing techniques, the mobility of ultrathin solution-cast assembled two-dimensional crystals was shown to be thickness independent,^10^ and ultrathin spin-coated films resulted in a saturated mobility after 2–6 molecular monolayers, depending on film morphology.^11^

While some spread is present in the experimental data, there is consensus that charge transport is happening predominantly in the first 2–3 monolayers from the dielectric interface. However, the analysis of these experiments is complicated by the dependence of the film morphology on semiconductor thickness and the detailed carrier distribution between these layers remains difficult to probe. The specific carrier distribution is an input for full transistor models, such as the Vissenberg-Matters model that has been successfully used to describe charge transport in organic FETs.^12^

The Vissenberg-Matters model is based on percolation hopping in an exponential density of localized states (DOS). The model yields a conductivity that increases as a function of carrier density and temperature. In order to arrive at an analytical expression for the source-drain current, Vissenberg and Matters use a carrier density profile in the accumulation layer that decreases quadratically with distance from the dielectric, a distribution referred to as 3D (**Figure** **1**, blue profile). Recently, Brondijk et al. analysed the carrier distribution in self-assembled monolayer transistors (SAMFETs) comprising a single layer of semiconductor only 2 nm thick.^13^ Brondijk et al. developed an analytical model based on a carrier distribution profile in the transistor accumulation layer where the carrier density is constant up to a certain thickness and zero further away from the dielectric, a profile termed 2D (Figure 1, yellow profile). The rationale for this carrier distribution profile in the SAMFET is that there is no space in a 2 nm thick semiconductor to generate the 3D carrier distribution profile. By using a Taylor expansion of the full expression of the source-drain current in the linear regime, the gate voltage dependence of the transistor current in this regime could be approximated by a power-law. A different exponent is found for the 2D and 3D case and the specific variation of the exponent as a function of temperature is utilized to distinguish between the two cases. From the distinction Brondijk et al. conclude that in Si/SiO_2_ bottom-gate polymer field-effect transistors – based on various semiconductors – the 3D carrier distribution applies. In contrast, in the SAMFET and monolayer transistors of evaporated 6T molecules the 2D case is appropriate.

**Figure 1:**
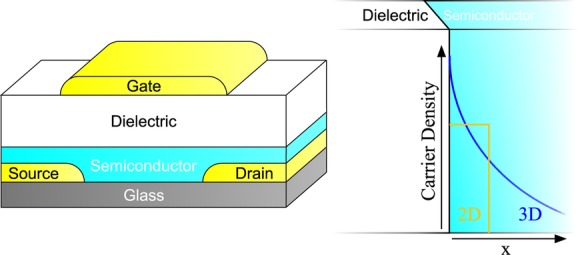
Schematic representation of the transistor geometry and the 2D and 3D carrier distribution profile in the accumulation layer of the transistors.

Brondijk et al. used a Taylor expansion to obtain an approximate power-law dependence of the source-drain current in the linear regime. In contrast, here we propose to analyse the charge transport in the saturation regime of the transistors. The source-drain current in the saturation regime is mathematically easily derived by substituting *V*_D_ with *V*_G_–*V*_t_.^14^ Adopting Equations (5) and (8) by Brondijk et al. and performing the aforementioned substitution leads to the simplified expressions for the source-drain current in the saturation regime:

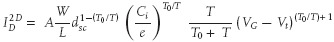
1

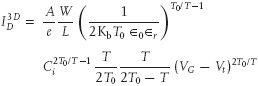
2 with

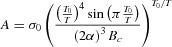
3

In the above expressions, *T*_0_ is the characteristic width of the exponential DOS, α^−1^ the wavefunction overlap localization length, σ_0_ a conductivity prefactor and *B*_c_ the critical number for the onset of percolation. The parameter *d_sc_* in the 2D case is the thickness of the slab in the semiconductor in which the constant carrier density resides. Significantly, as seen from Equations (1) and (2), performing the analysis in the saturation regime eliminates the need for a Taylor expansion to obtain an approximate power-law dependence of the source-drain current on gate voltage. In the saturation regime, both the 2D and 3D based model lead to direct power-laws *I*_D_ ∝ (*V*_G_ – *V*_t_)^γ^, with γ_2D_ = (*T*_0_/*T*) + 1 and γ_3D_ = 2*T*_0_/*T*. Analysing the value of the power γ as a function of temperature therefore allows us to discern between a 2D and 3D distribution. Additionally, the value of *T*_0_ can be extracted from the slope of γ versus 1/*T*.

We analyse standard p- and n-type top-gate polymer FETs with interdigitated Au source and drain electrodes using a range of polymers semiconductors. We fabricated transistors using poly([2,5-bis(2-octyldodecyl)-2,3,5,6-tetrahydro-3,6-dioxopyrrolo[3,4-c]pyrrole-1,4-diyl]-*alt*-[[2,2′-(2,5-selenophene)bis-dithieno(3,2-b;2′,3′-*d*)thiophene]-5,5′-diyl]) (PSeDPPDTT),^15^ poly(3-hexylthiophene) (P3HT), poly([N,N′-bis(2-octyldodecyl)-naphthalene-1,4,5,8-bis(dicarboximide)-2,6-diyl]-*alt*-[5,5′-(2,2′-bithiophene)]) (N2200) and poly(2,5-bis(3-tetradecylthiophen-2-yl)thieno[3,2-b]thiophene) (PBTTT) in combination with a PMMA dielectric, and poly(bis(4-phenyl)(2,4,6-trimethylphenyl)amine) (PTAA) in combination with a CYTOP dielectric. The semiconductor layers were spin-coated from solution, yielding layer thicknesses ranging from 60–100 nm, significantly thicker than the expected accumulation layer thicknesses. Transistor transfer characteristics were measured in the saturation regime (|*V*_D_| = 60 V) as a function of temperature in a vacuum probe station.

**Figure** **2**a displays the measured transfer characteristics of FETs based on PSeDPPDTT as a function of temperature. The presented characteristics are representative for all fabricated transistors and extracted room-temperature mobility values and Arrhenius-type activation energies are summarized in **Table** **1**. As a first step in the further analysis of the charge transport, Figure 2b replots the transfer characteristics on a double logarithmic scale. Straight lines are obtained for all temperatures indicating a power-law dependence of the source-drain current as a function of gate bias. The exponent γ of the power-law is determined from the transfer curves for all distinct temperatures and is plotted in Figure 2c versus 1/T to conclude the simple analysis. A straight line is obtained once more. The extrapolated linear fit intersects with the vertical axis at a value of 1, indicative of the fact that the transfer characteristics are in accordance with Equation (1). This leads to the conclusion that the carrier distribution profile in the PSeDPPDTT top-gate transistors is two-dimensional.

**Table 1:** Parameters of the fabricated transistors: conduction type, extracted room-temperature mobility μ_300K_, activation energy *E*_a_, width of the semiconductors DOS *T*_0_ and Urbach energy *E*_u_

Semiconductor/Dielectric	Conduction	μ_300K_ (cm^2^ V^-1^s^-1^)	*E*_a_ (meV)	*T*_0_ (K)	*E*_u_ (meV)
PSeDPPDTT/PMMA	p-type	0.09	135	445	39
N2200/PMMA	n-type	0.15	98	366	32
P3HT/PMMA	p-type	0.07	123	585	50
PBTTT/PMMA	p-type	0.06	162	670	58
PTAA/CYTOP	p-type	0.003	183	453	39

**Figure 2:**
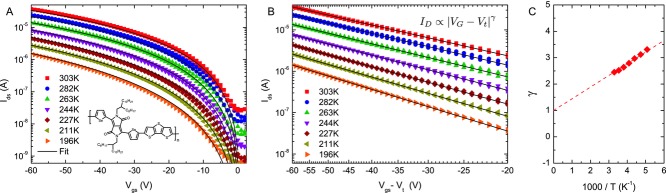
(a) Experimental transfer characteristics of a PSeDPPDTT-based FET (L = 20 μm, W = 1000 μm) as a function of temperature. Solid lines are fits to Equation (1). (b) Replotted transfer characteristics of (a) on a double logarithmic scale. Solid lines are fits to extract the parameter γ for each temperature. (c) Extracted values of γ from (b) plotted versus 1/T. The extrapolated dashed linear fit yields the intersection with the vertical axis while the value of *T*_0_ is derived from the slope.

**Figure** **3** shows the constructed γ versus 1/T graphs for the other materials investigated in this work. All graphs show a vertical axis intersection at a value of 1, demonstrating the 2D carrier distribution profile is omnipresent in all fabricated polymer top-gate transistors, independent of the type of carrier (p-type vs. n-type) and specific dielectric used (PMMA vs. CYTOP). The original 3D Vissenberg-Matters expression, which would result in a vertical axis intercept of γ = 0, can therefore not be used to properly describe charge-transport in our top-gate polymer FETs. Rather, the solid lines in Figure 1a show a good fit of Equation (1) to the transfer characteristics. This is representative for all fabricated transistors.

**Figure 3:**
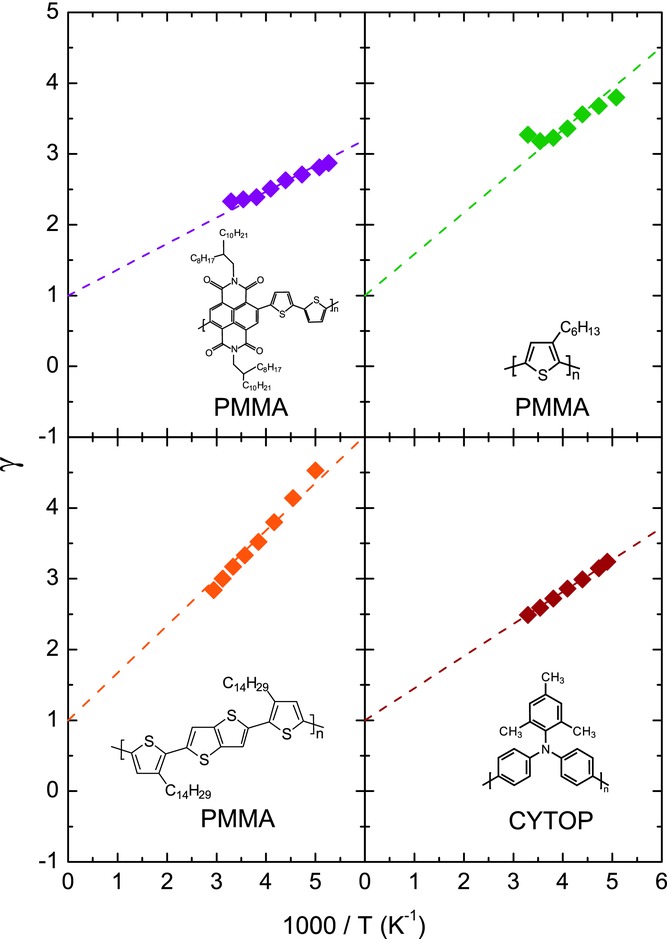
Extracted values of γ plotted versus 1/T for FETs based on, from top-left to bottom-right: N2200 (n-type), P3HT, PBTTT and PTAA. The insets show the molecular structure of the polymer semiconductor and indicate the respective dielectric used for the FETs. The 2D nature of the charge distribution is confirmed by the vertical-axis intersection at γ = 1 and the value of *T*_0_ is derived from the slope.

The slopes of the linear fits in the γ versus 1/T graphs yield the value of T_0_, the characteristic width of the (exponential) DOS. In order to verify the extracted T_0_ values we set out to obtain an additional independent measurement that is sensitive to the amount of disorder in the polymer films. In inorganic semiconductors, e.g., α-Si, disorder is known to result in the formation of states in the bandgap, broadening of the absorption onset and creating an exponential sub-bandgap absorption tail called the Urbach tail.^16^ The characteristic width of the exponential absorption tail *E_u_*, i.e., the Urbach energy, has been shown to correlate with the amount of disorder in the material.^17^ In order to obtain the Urbach energies for the polymer semiconductors, we performed Photothermal Deflection Spectroscopy (PDS) on spincoated thin films. PDS is a sensitive spectroscopic technique able to accurately measure weak absorptions in the bandgap.^18^,^19^ **Figure** **4** shows the normalized PDS absorption spectra for the polymer films. All polymer films show an extended region below the bandgap where the absorption of the films decreases exponentially and from which values of the Urbach energy were extracted. A large error exists in the exact determination of the Urbach energy by virtue of the relatively arbitrary selection of the width of the exponential region to fit. Using different fitting procedures we have estimated the error in the determination of the Urbach energy. It is, nevertheless, possible to distinguish differences in the Urbach energies among the investigated polymers. N2200 has the sharpest bandtail while P3HT and PBTTT have significantly wider bandtails. This is consistent with the low degree of energetic disorder that has previously been reported for N2200.^20^

**Figure 4:**
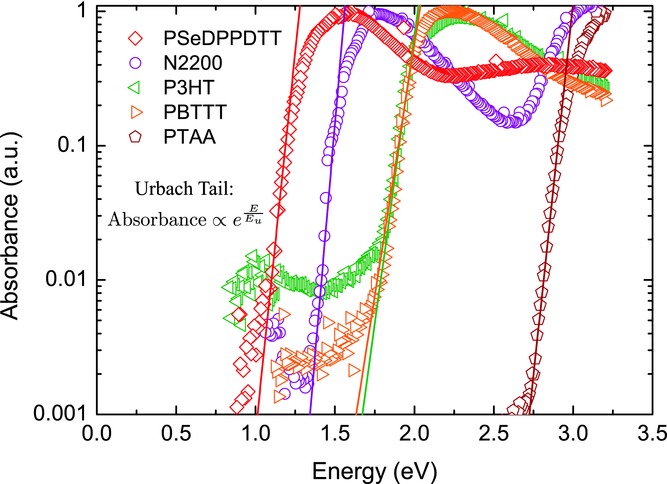
Normalized absorbance of spincoated polymer thin films measured by Photothermal Deflection Spectroscopy. Solid lines show fits to the exponential sub-bandgap regions using *E*_u_ equal to the extracted *T*_0_ values from the 2D FET model.

**Figure** **5** plots the *T*_0_ values as determined from the temperature-dependent FET measurements versus the extracted Urbach energy from the PDS measurements. In spite of the errors in the values of the Urbach energy a convincing case for a linear proportionality between the two parameters can still be made. This is not unexpected since both parameters are measures of the degree of energetic disorder in the films. The proportionality gives us confidence that the extracted *T*_0_ values have physical relevance and supports the above conclusion that the two-dimensional model is more appropriate to describe charge transport in top-gate polymer FETs than the three-dimensional model.

**Figure 5:**
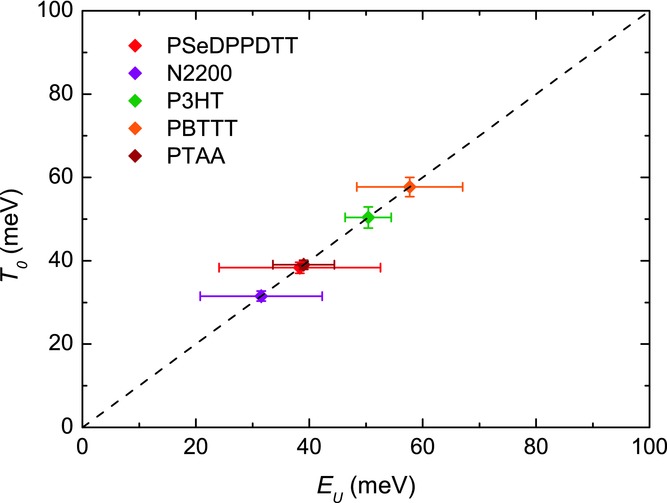
Correlation between the width of the DOS extracted from the FET transfer characteristics and the Urbach energy extracted from the PDS absorption spectra.

What is more surprising is that the Urbach energy extracted from the PDS measurements appears in fact to be identical to the width of the exponential DOS extracted from the 2D Vissenberg-Matters model fits, i.e., *E_u_ = k_B_ T_0_*. The solid line fits in Figure 4 use fixed values of *E_u_*, *= k_B_ T_0_* as extracted from the transport measurements. They provide very good agreement with the PDS absorption tails. This agreement is not necessarily expected in an organic semiconductor, since the lowest optical absorption band is of an excitonic nature and neutral excitons are expected to be less strongly affected by certain types of disorder, such as electrostatic (dipolar or quadrupolar) disorder than charge carriers. This might suggest that there is an interplay between dipolar disorder and other sources of disorder that affect neutral excitons more than charge carriers, such as variations in bandgap due to conformational defects and variations of conjugation length of the polymer backbone. This question will be the subject of further investigations.

Returning to the 2D model, we would like to comment on the fitting of the complete transfer characteristics using the 2D model as shown in Figure a. With the *T*_0_ values for the different polymers determined from the aforementioned analysis, Equation (1) contains three additional parameters to describe the complete transfer characteristics of the FETs. The three parameters, i.e., α^−1^, *d_sc_* and σ_0_, are to be determined. We found, however, that there is no unambiguous set of values for these parameters that could be determined from the fit shown in Figure 1a. Over a wide range of values the parameters α^−1^ and *d_sc_* are interchangeable in order to match the temperature dependence of the charge transport, with the parameter σ_0_ available to adjust the absolute value of the source-drain current for a particular combination of α^−1^ and *d_sc_*. **Figure** **6** illustrates the parameter space for which identical quality fits to the experimental data are obtained. Relationships between the parameters are observed that are in accordance with Equation (1). In the present case of top-gate FETs with relatively thick semiconductor layers it is not possible to fix *d*_*sc*_ a priori, in contrast with the case of the SAMFET where the thickness of the semiconductor is known and set as the accumulation layer thickness. For (semi)crystalline polymer films with edge-on, lamellar orientation, such as P3HT or PBTTT, it is tempting to assume the thickness of the first layer of polymer chains in the unit cell to define the accumulation layer thickness *d_sc_*. We have therefore constrained the analysis to *d*_sc_ ≤ 2 nm. In amorphous polymers or polymers with face-on orientation it is less clear, a priori, how to determine a unique value for *d*_sc_.

**Figure 6:**
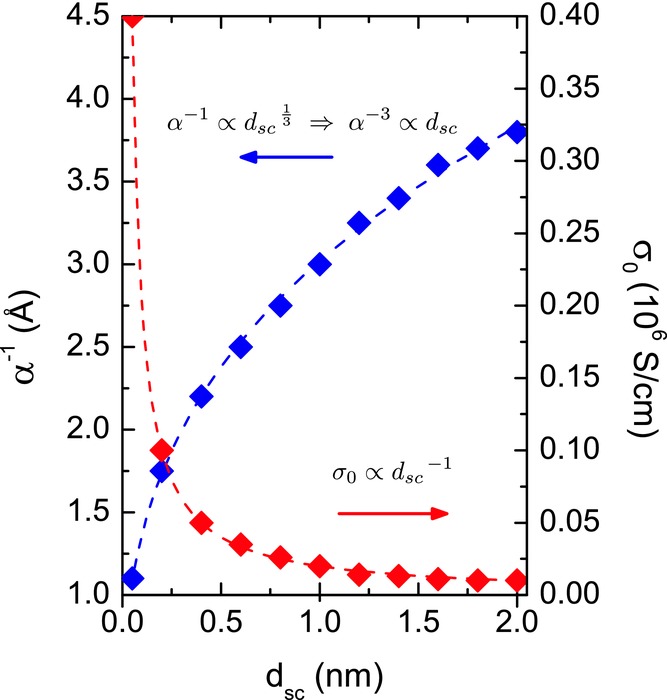
Visualisation of the parameter space that yields identical fits of the temperature-dependent transfer characteristics of PSeDPPDTT FETs using Equation (1). The resulting fits are shown as solid lines in Figure 1a. A threshold voltage V_t_ = +1 V was used. Dashed lines are a guide to the eye showing expected relationships between the parameters from Equation (1).

Our results demonstrate that in top-gate polymer FETs the charge distribution in the accumulation layer is best modelled as two-dimensional even under conditions where the semiconductor layer is clearly thicker than the accumulation layer. This is in contrast with the results obtained by Brondijk et al., who found that bottom-gate Si/SiO_2_ transistors with thick polymer films are best described by the three-dimensional model. The reason for this discrepancy is not clear at present. The analysis of Brondijk et al. is performed in the linear regime in contrast to the current analysis, which is based on the saturation regime. However, we believe the charge carrier distribution in the accumulation layer of the transistors will be independent of the operating regime of the field-effect transistor. A possible reason for the discrepancy could be the different polymer semiconductors and dielectrics used. The semiconductors used by Brondijk et al. exhibit significantly lower mobilities, even when compensated for the fact that the obtained mobilities are extracted in the linear regime instead of the saturation regime. The lower mobilities are a possible indication of bottlenecks in carrier transport along the dielectric interface and of a higher degree of disorder. In addition, random dipoles in dielectrics with higher dielectric constant or polar surface groups, such as SiO_2_, are known to induce additional dipolar disorder in the semiconductor at the interface.^21^–^23^ Bottlenecks along the interface in conjunction with an increased effect of interfacial (dipolar) disorder might force charge carriers further away from the dielectric interface resulting in a 3D carrier distribution. In contrast, the higher mobilities that are obtained for the current semiconductors indicate that bottlenecks for charge transport are less of importance while the above close correlation between Urbach energy (which is extracted from a bulk spectroscopic measurement) and *k_B_ T_0_* (which is extracted from an interfacial transport measurement) does indeed suggest that in our top-gate FETs with polymer dielectrics of relatively small dielectric constant interfacial dipolar disorder is not strong. We emphasize, however, that in preliminary measurements of bottom-gate bottom-contact transistors with the materials investigated here we have not observed the expected 3D characteristics. This is an additional indication that the carrier distribution may be related to the specific polymer semiconductors used and this will be the subject of further investigation.

In conclusion, we have investigated the charge carrier distribution in the accumulation layer of top-gate polymer field-effect transistors by analysing the transfer characteristics in the saturation regime as a function of temperature using a Vissenberg-Matters hopping model framework with 2D and 3D carrier distribution profiles. By comparing experimental transfer characteristics of various top-gate polymer field-effect transistors with model predictions, a general semiconductor-independent two-dimensional character of the carrier distribution in the accumulation layer of these types of transistors is revealed. The results are corroborated by the extraction of Urbach energies from the sub-bandgap absorption tails of the polymer semiconductors that are identical to the T_0_ values extracted from the 2D carrier distribution model. The correlation between the width of the DOS and the Urbach energy is a significant result in itself, demonstrating an intricate connection between optical measurements concerning disorder and disorder effects on charge-transport in organic FETs. The results show that the original Vissenberg-Matters model cannot be used to describe charge transport in top-gate polymer FETs and the adapted model taking into account the 2D carrier distribution needs to be applied. Although the parameter T_0_ can be directly determined, within the adapted model framework an unambiguous set of values for the other parameters describing charge transport cannot yet be obtained, since the accumulation layer thickness cannot be set a priori as a fixed parameter. This will require further refinement of the model. Our results open up new potential to advance the understanding of charge transport in polymer FETs, investigate sources of disorder in order to progress the carrier mobility in polymer semiconductor films and extract more reliable values for fundamental charge transport parameters of polymer transistors to obtain direction to beneficially adjust device performance.

## Experimental Section

Bottom-contact top-gate transistors were fabricated with PMMA or CYTOP polymer gate dielectrics. Gold source and drain bottom electrodes (with Cr as the adhesion layer) were patterned by photolithography on clean glass substrates. Thin films of the polymer semiconductors were deposited on top by spin coating, resulting in layer thicknesses of 60–100 nm. Subsequently, PMMA or CYTOP was spin coated onto the semiconductors. Finally, gold gate electrodes were evaporated by shadow mask evaporation. Transistor transfer characteristics were measured with an Agilent 4155B Semiconductor Parameter Analyser in the saturation regime (|*V*_D_| = 60 V). Temperature-dependent measurements were performed in a Desert Cryogenics vacuum probe station cooled with liquid nitrogen.

Photothermal Deflection Spectroscopy (PDS) was performed on identically prepared polymer films. PDS is a sensitive absorption measurement technique that makes use of the heating effect produced due to the non-radiative relaxation of the states excited by the absorption of electromagnetic radiation. The produced heat generates a thermal gradient in the space surrounding the sample surface, resulting in a refractive index gradient which is proportional to the absorbed light in the sample. In order to amplify the refractive index gradient the samples were held in a hermetically sealed fused silica cuvette filled with an inert liquid-Fluorinert FC-72 (3M Corporation). This liquid shows a high refractive index change for a small change in temperature and acts as a deflection medium. The PDS setup used is similar to that described by Jackson et al.^19^ Samples were excited using a modulated monochromated light beam, produced by a combination of a Light Support MKII 100 W Xenon arc source and a CVI DK240 monochromator, inclined perpendicular to the plane of the sample. A transverse probe beam, produced using a Qioptiq 670 nm fiber-coupled diode laser with temperature stabilizer for reduced beam pointing noise, was passed through the thermal gradient in front of the sample surface. Beam deflections, caused by the absoption of light in the sample and the creation of a refractive index gradient in front of the sample surface, were measured using a differentially amplified quadrant photodiode and a Stanford Research SR830 lock-in amplifier.
